# Activity-controlled annealing of colloidal monolayers

**DOI:** 10.1038/s41467-019-11362-y

**Published:** 2019-07-29

**Authors:** Sophie Ramananarivo, Etienne Ducrot, Jeremie Palacci

**Affiliations:** 1 Department of Physics, University of California San Diego, 9500 Gilman Drive, La Jolla, CA 92093-0319 USA; 2 Center for Soft Matter Research, Department of Physics, New York University, 726 Broadway, New York, NY 10003 USA; 3 Present Address: Ladhyx, Ecole Polytechnique, Institut Polytechnique de Paris, 91128 Palaiseau Cedex, France

**Keywords:** Colloids, Self-assembly, Statistical physics, thermodynamics and nonlinear dynamics, Soft materials

## Abstract

Molecular motors are essential to the living, generating fluctuations that boost transport and assist assembly. Active colloids, that consume energy to move, hold similar potential for man-made materials controlled by forces generated from within. Yet, their use as a powerhouse in materials science lacks. Here we show a massive acceleration of the annealing of a monolayer of passive beads by moderate addition of self-propelled microparticles. We rationalize our observations with a model of collisions that drive active fluctuations and activate the annealing. The experiment is quantitatively compared with Brownian dynamic simulations that further unveil a dynamical transition in the mechanism of annealing. Active dopants travel uniformly in the system or co-localize at the grain boundaries as a result of the persistence of their motion. Our findings uncover the potential of internal activity to control materials and lay the groundwork for the rise of materials science beyond equilibrium.

## Abstract (web summary)

Active particles added to a material control its internal activity. Here the authors investigate such a system and control the annealing of a colloidal crystal, the dynamics of which depend on the persistence of the motion of active particles.

## Introduction

In the classical picture of Brownian motion, particles and fluid molecules are passive and driven by thermal fluctuations^1–3^. The incessant motion of suspended particles is then due to multiple collisions with the surrounding fluid molecules. In living systems, molecular motors ballistically zoom around, push and pull on each others, and exert forces on passive elements, yielding a riotous environment radically different from the more mundane equilibrium one. This non-equilibrium dynamics induces additional fluctuations^4,5^ that boost transport in biological systems^6,7^ or quickly bring building blocks into contact while reducing unwanted connections^8^. The effect of internal agitation has alternatively been discussed in the different context of driven vortex lattices in high-temperature superconductors^9,10^. Active colloids, made available by recent synthetic progress^11^, similarly inject energy locally and drive systems out of equilibrium. Yet, developments in Active Matter have primarily focused on exotic behavior without equilibrium counterpart: broken microscopic reversibility^12,13^, and mesoscopic fluxes such as flocks^14^, flows^15,16^, phase separation^17–20^, or anomalous transport of dispersed tracers^21–23^. It largely overlooked their practical impact to deploy materials science beyond equilibrium and control matter. Active particles added to a material generate forces from within and provide a unique opportunity to overcome the naturally occurring kinetic barriers and modulate the energy landscape of soft materials. This potential was highlighted in numerical studies of a single colloid driven through a colloidal crystal^24^ that showed local melting, generating lattice defects that increase the drag force and induce large noise fluctuations^24^. Opportunities in colloidal assembly were further stressed by recent numerical works that considered mixtures of active, autonomously-driven particles, in passive phases. They showed that the presence of active particles can favor the annealing of a passive polycrystal by melting its grain boundaries^25–27^ or act as an internal field to control the structural and dynamical properties of a colloidal gel^28^. Notwithstanding, experimental realizations of mixtures of active particles in a passive phase are scarce and have not demonstrated control of the passive phase. Dietrich et al.^29^ showed that the motion of self-propelled particles is altered by the presence of a loosely packed crystal and focused on the dynamics of the active individuals. Alternatively, Kummel et al.^27^ showed that in sparse colloidal layers, active particles tend to gather colloids into dynamical clusters, while in denser ones, they accumulate at the grain boundaries separating crystalline domains, where they initiate melting and widen those disordered regions. Although further numerical modeling of their system suggests that this localized melting could lead to a large defect-free crystal^27^, the exploitation of active dopants as a workhorse for material design remained to be achieved experimentally.

In this work, we devise a microfluidic system of confining arenas with slanted walls, that allows exchange of active particles with an external reservoir and enables a uniform injection of energy to the passive phase. It makes possible the control of the annealing of a colloidal monolayer in time and space by harnessing the activity of embedded active intruders, offering the first experimental demonstration of man-made materials internally controlled by active noise.

## Results

### Accelerated annealing

**Fig. 1:**
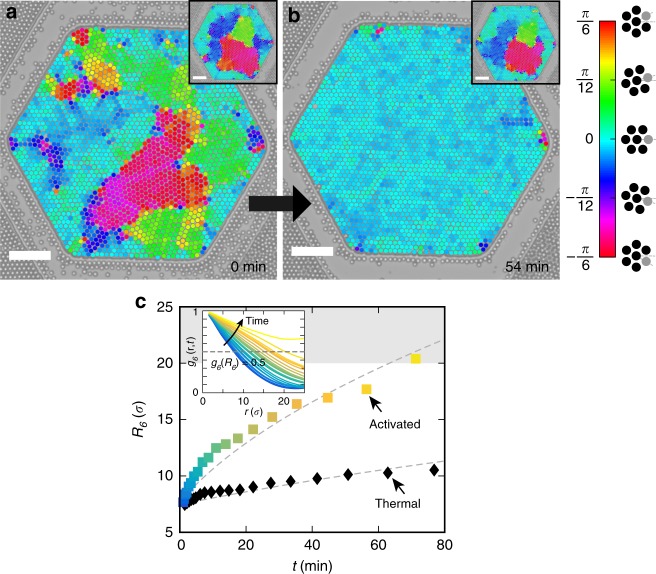
Accelerated annealing. **a**, **b** Optical microscopy of a hexatic monolayer of passive beads (circles) containing a small fraction of active intruders (not visible at this magnification). The passive beads are color-coded by the local orientational field of the hexatic order. **a** The system is initially quenched in adjacent polycristallites and **b** rapidly relaxes towards an ordered monolayer, following the activation of the self-propelled particles in the layer. The thermal experiment, in the absence of active particles, is significantly slower and shows little evolution within the same time-span (insets). Scale bars, 50 μm. **c** (inset) Bond orientational correlation function *g*_6_(*r*, *t*) as a function of distance *r* and for increasing times *t* (blue to yellow color gradient) (see main text). It increases with time as long-range order develops: large grains grow at the expense of smaller ones. **c** Time-evolution of the characteristic grain radius *R*_6_, in units of colloid diameters *σ*, defined from *g*_6_(*R*_6_, *t*) = 0.5 for thermal (black) monolayer or in the presence of embedded self-propelled particles (color, *V* = 10 μm s^−1^, *α* = 1.3%) and fit by a normal grain-growth *R*_6_(*t*)^2^ = *R*_6_(0)^2^ + *γt* (dotted lines, see Main text). The gray area delimits grain sizes >20*σ*, which cannot be measured accurately in the experiment

Our experimental system consists of a suspension of beads of diameter *σ* = 5 μm (Sigma aldrich, silicon dioxide, 44054) filling a hexagonal well embossed on the bottom substrate of a microfluidic chamber. After sedimentation, the beads form a dense monolayer of spheres at a surface fraction Φ_S_~0.68 ± 0.03 with a local hexatic order (Methods and Supplementary Fig. 1). The rapid increase of the surface density of the sedimenting particles quenches the system into adjacent crystallites separated by grain boundaries (Fig. 1a), a metastable state that eventually converts into a single crystal. At thermal equilibrium, it takes about 12 h for a system of 2200 beads to form a single crystal in a hexagonal chamber of width 260 μm. We explore the impact of the addition of a small fraction of active intruders to this initially polycrystalline monolayer of silica spheres. The active intruders are light-activated microswimmers, consisting of a hematite (Fe_2_O_3_) portion protruding out of a polymer sphere^20^; they are 2 μm in diameter and exhibit a two-dimensional persistent random walk in free space^30^. The iron oxide photocatalytically decomposes a solution of hydrogen peroxide fuel after activation by a wavelength *λ* = 390–480 nm, resulting in subsequent propulsion of the particle in the concentration gradient^20^. The velocity is controlled by the intensity of the light^20^ that illuminates the microfluidic chamber uniformly. We periodically interrupt the propulsion of the active particles by short periods of extinction, light off, to allow the swimmers possibly wedged between the passive spheres and the substrate to reorient and be released. We observe a qualitative difference of dynamics between thermal and active annealing. In the latter, streaks form following the displacement of the intruders in the crystal (Supplementary Movie 1). Within 40 min, the activated system reorganizes towards a fully ordered state (Fig. 1b), whereas its thermal counterpart—with no swimming activity—has undergone little change (Fig. 1a, b-insets and Supplementary Movie 1).

To gain insight into the phenomenon, we vary the numeric fraction *α* of intruders in the monolayer, *α* = 0.1–5% of the total number of passive beads, and their propulsion velocity *V* using the control offered by light. We perform the experiments in larger systems with 5600 passive beads, for which the reorganization can be followed on larger spatial and temporal scales. The passive beads are gravitationally confined in a hexagonal arena, whose slant and limited depth *h* ~ 4 μm, allow the self-propelled particles to escape and enter^31^. This avoids the accumulation of the active particles at the walls and maintains a constant surface fraction Φ_S_ of passive beads and swimmers, an important prescription to the present study of annealing.

We quantify the temporal and spatial properties of the monolayer using the bond-orientational correlation function *g*_6_(*r*, *t*), calculated from the sixfold bond-order parameter (see Methods and Supplementary Fig. 1). The function *g*_6_(*r*) increases with time as long-range order develops (Fig. 1c-inset): the system coarsens and large grains develop at the expense of the smaller ones with the walls favoring crystallites that are geometrically compatible with the boundaries (Supplementary Movie 2). A characteristic grain size *R*_6_ is set by the threshold of the bond-orientational correlation function, *g*_6_(*R*_6_, *t*) = 0.5 (Fig. 1c) as previously introduced in thermal systems^32,33^, and confirmed as a relevant length scale of the grain structure by the time-independent rescaling *g*_6_(*r*, *t*) = *f*(*r*/*R*_6_(*t*)) (Supplementary Fig. 2). The grain size *R*_6_(*t*) increases with time and shows a reasonable agreement with the normal law for a curvature-driven grain-growth, d*R*_6_/d*t* = *γ*/*R*_6_, where *γ* is the mobility of the grain boundary^34^ (Fig. 1c). It offers a convenient observable to quantify the large speeding up of the annealing (see SI). Devising models that capture more finely the scaling of the coarsening mechanisms in out-of-equilibrium systems, constitutes an exciting development, which is beyond the scope of the present work. We further use *γ* as a measure of the observed dynamics and study its dependence on the speed and the fraction of active intruders. We report a massive increase in *γ*, up to 40-fold, compared to a thermal system.

### Microscopic dynamics and activated process

**Fig. 2:**
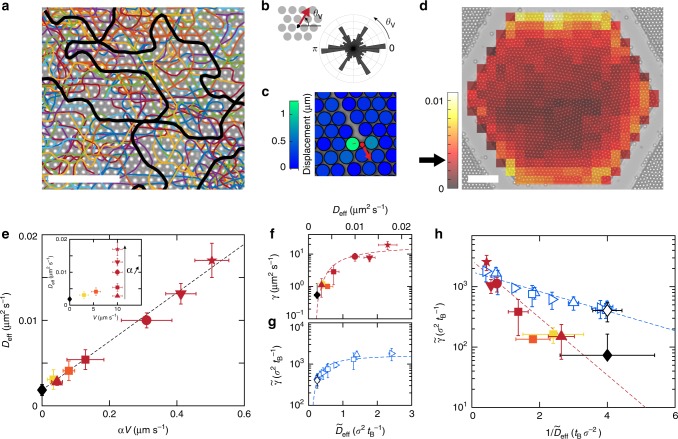
Microscopic dynamics. **a** Individual trajectories of self-propelled particles in an ordered monolayer. Some trajectories are highlighted in black for better visibility. **b** The probability distribution of propulsion direction *θ*_V_ of the active particles shows peaks along the natural directions of the crystal. **c** Displacement of colloids induced by a swimmer (red dot, the arrow indicates the direction of motion); the color-coding of particles denotes the amplitude of displacement (traced by red lines) following a collision. **d** Probability distribution of the presence of swimmers in the colloidal layer over the first 20 min of an experiment. It is uniform (value for a uniform distribution indicated by an arrow), the swimmers do not localize on the walls, the grain boundaries or elsewhere. Scale bars, 50 μm. **e** (inset) Measured diffusivity *D*_eff_ of the passive colloids of the monolayer as a function of the speed *V* of the active particles. Colors distinguish speeds *V* = 3 (yellow), 5 (orange), 10 μm s^−1^ (dark red); and symbols refer to different fraction of swimmers *α* = 0.4 (upward triangle), 1.3 (square), 3.1 (circle), 4.1 (downward triangle), and 5.0% (star). **e** The data collapse onto a master curve *D*_eff_ = D^*^ + *βαV* when plotted as a function of *αV*, with D^*^ the diffusivity for a purely passive system (black diamond), simply described by a model of collisions of the passive beads with the active particles (see main text). **f** Grain mobility *γ* as a function of *D*_eff_ in experiments; the presence of intruders speed up the reorganization of the monolayer by nearly two orders of magnitude. **g** *γ*(*D*_eff_) obtained in simulations by varying the velocity of self-propelled particles at a fixed persistence time comparable with the experiments (see Methods). Data are nondimensionalized using *σ* and the Brownian time *t*_B_ as a characteristic length and time (see main text); symbols differentiate between fractions of swimmers *α* = 0.3 (upward triangle), 0.6 (right-pointing triangle), and 1.2% (square). **h** Log-lin plot of *γ* as a function of 1/*D*_eff_ shows an Arrhenius-like behavior in the experiment (color symbols) and the simulations (hollow symbols)

In order to connect the observed phenomenon with the individual dynamics of the particles, we follow the trajectories of the self-propelled particles in the well. They travel along the local directions of the surrounding crystal (Fig. 2a–c), with a speed *V* = 3–10 μm s^−1^, controlled by light, and a persistence time of *τ*_R_ ~ 2 s comparable with the values measured in free space (Supplementary Fig. 4). The trajectories show no apparent directional persistence from one crystallite to the next, as particles reorient upon crossing a grain boundary. Swimmers travel uniformly in the chamber with limited accumulation at the walls and show no significant localization at the grain boundaries or elsewhere (Fig. 2d). This result contrasts with previous numerical reports of intruders accumulating at the grain boundaries and initiating the melting from the surface^25,26^. It further confirms the absence of motility-induced phase separation^35,36^ that would notably arise from a non-uniform illumination.

We further quantify the impact of the activity by tracking the individual trajectories of the passive beads. The dynamics are complex and depend on local rearrangements and instantaneous interactions with the self-propelled particles. In virtue of the uniform distribution of the self-propelled particles (Fig. 2d), we adopt a coarse-grained approach and consider an ensemble average of the passive particles during the first 20 min of the experiment. The dynamics are diffusive at long times and we extract the effective diffusion coefficient *D*_eff_ from the linear fit of the mean square displacement (Supplementary Fig. 3). Experimental results for varying speeds and fraction of swimmers (Fig. 2e-inset) gather on a master curve: *D*_eff_ = D^*^ + *βαV*, where D^*^ is the diffusivity at equilibrium measured independently and *β* ~ 3.10^−2^ μm, the single fitting parameter for the experimental data (Fig. 2e). We rationalize this result by a kinetic model of intruders colliding with the monolayer, similar in spirit to Mino et al.^23^ in the opposite limit of dilute tracers in an active bath. Considering the passive beads as fixed targets, their decorrelation *τ* time is set by the inverse of the collision rate with the active particles, $\tau^{-1} {\propto} \phi _{\mathrm{a}}V{\ell}$, where $\phi _{\mathrm{a}}\sim \alpha {\mathrm{\Phi}}_{\mathrm{S}}/\sigma ^{2}$ is the number of intruders per unit area, and $\ell$ is the interaction length transverse to the motion of the swimmers. Each collision induces a displacement *d*, independent of the velocity *V* at low Reynolds number, so that the active contribution to the diffusivity of the beads scales as $d^{2}/\tau {\propto} \frac{d^{2}\ell {\mathrm{\Phi}}_{\mathrm{S}}}{\sigma^{2}}\alpha V$. Comparison with the experiment gives $\frac{d^{2}\ell {\mathrm{\Phi }}_{\mathrm{S}}}{\sigma ^{2}}\sim 3.10^{ - 2}\,{\upmu} {\mathrm{m}}$. This leads to $d^{2}\ell \sim 1\,{\upmu} \mathrm{m}^{3}$, that is $d\sim \ell \sim \sigma /5$, in line with experimental observations (Fig. 2c) and a simple scenario dominated by hard-sphere collisions without entrainment.

Next, we connect the grain mobility *γ* with the behavior of the passive beads in the monolayer. The grain-growth increase correlates with the enhancement of the diffusivity (Fig. 2f) and follows an Arrhenius-like law, *γ* ∝ exp − *A*/*D*_eff_ (Fig. 2h). Active fluctuations that originate from the presence of the self-propelled particles allow to cross the energy barrier *E* required to displace grain boundaries. We extract from the data, $A\sim E/\mu ^{\ast} \sim 8.10^{ - 3} \pm 1.10^{ - 3}\,{\upmu} \mathrm{m}^{2} \mathrm{s}^{-1}$, where μ^*^ is the equilibrium mobility relating equilibrium fluctuation and dissipation^37^, D^*^ = *kT*/μ^*^, larger than the Stokes mobility due to additional dissipation from confinement. It gives $E\sim 4 \pm 1\,kT$, a reasonable value for the entropic barrier of a dense monolayer of hard spheres.

### Numerical simulation of a colloidal monolayer

**Fig. 3:**
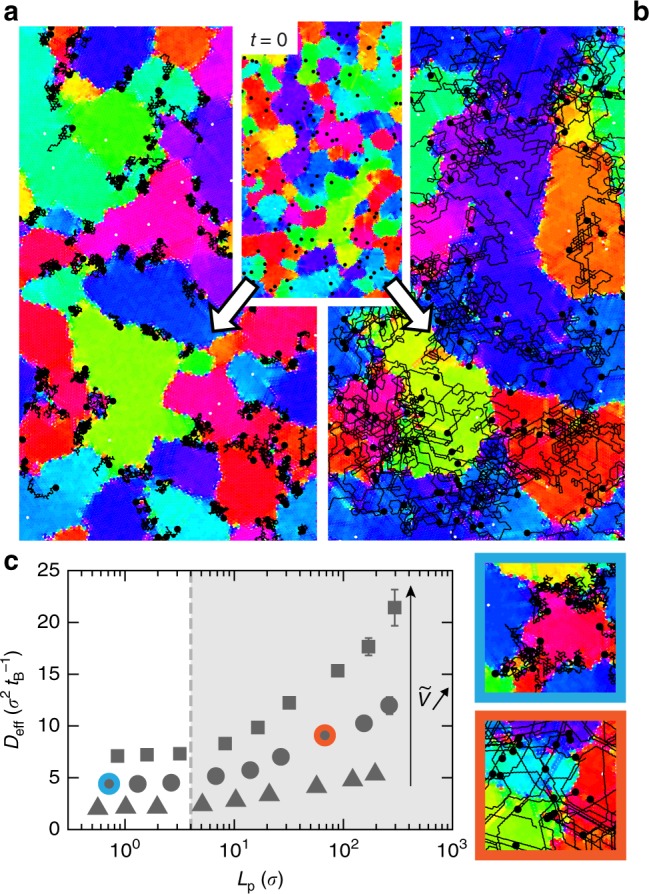
Numerical simulation of a colloidal monolayer, with added active particles of different persistence. Evolution of the system starting from the same initial configuration (inset, *t* = 0) with a fraction *α* = 0.6% of active intruders, with same speed but different persistence time, leading to persistence lengths of **a** *L*_p_ = 0.3*σ*, and **b** *L*_p_ = 7*σ* comparable to experiments. The dynamics of the swimmers change qualitatively with the persistent time: they navigate along the directions of the crystal in one case (**b**) and localize at the grain boundaries in the other (**a**). The colors correspond to the local hexactic orientational order as in (Fig. 1). The active particles are shown as black points bigger than their actual size to improve visibility, and black lines trace their trajectories within the 3.10^6^ time steps preceding the snapshots. **c** The diffusivity *D*_eff_ of the passive particles shows a dynamic transition set by the persistence length *L*_p_ of the active dopants and plateaus for *L*_p_ ≤ 0.4*σ*. Active dopants co-localize at the grain boundary for *L*_p_ ≤ 0.4*σ* and travel in bulk otherwise, as illustrated with typical trajectories on the side panels, which correspond to the highlighted points on the graph. The qualitative change in the annealing mechanism, as visible on panels **a** and **b**, coincides with a transition in energy transfer between the dopants and the passive particles

We then compare our experiments with the results of two-dimensional Brownian dynamics simulations^38,39^ of a polycrystalline monolayer of 2.10^4^ particles, with Φ_S_ = 0.67, and periodic boundary conditions (Methods and Fig. 3a, b). A fraction *α* = 0.3 – 1.2% of active particles, with diameter 0.2*σ*, is pulled at a constant force and displaced into the monolayer. The persistence time of the motion can be modified to explore regimes which are not accessible to the experiment. For persistence times comparable with the experiment, the simulations qualitatively capture the behavior of the system: the intruders propel, following the local order of the crystallites. They form streaks in the monolayer (Fig. 3b) and speed up its annealing towards a long-range hexatic order (Supplementary Movie 3). We vary the amplitude of the pulling force and extract the velocity of the active particles, the mobility of the grain boundary and the effective diffusivity of the passive beads as in experiments. In order to compare experimental and numerical results, we express distances in bead diameters *σ* and time in diffusive time *t*_B_ = *σ*^2^/4D^*^: the time taken by a passive bead of the monolayer to diffuse one diameter *σ* in the absence of activity. The numerical relationship between the resulting dimensionless velocity $\tilde V$ and effective diffusivity $\tilde D_{{\mathrm{eff}}}$ deviates from that of experiments as a result of differences in momentum transfer between both systems: absence of hydrodynamics, dissipation with the substrate, or purely two-dimensional dynamics in simulations (Supplementary Note 4). However, the simulations and the experiments agree reasonably when comparing the dimensionless grain mobilities with the effective diffusivity of the passive beads, $\tilde \gamma (\tilde D_{{\mathrm{eff}}})$, (Fig. 2g), both showing an Arrhenius-like behavior. It confirms and highlights the origin of the phenomenon: active fluctuations resulting from the collision of the active beads with the passive matrix activate the process of annealing (Fig. 2h). Larger swimmers close in size to the passive beads, comparatively stall in the monolayer as their motion requires the formation of energetically costly disclinations, as previously reported by van der Meer et al.^25^ in simulations where all particles have identical sizes. Collisions with the passive matrix are largely reduced, and the effect of the active doping on the annealing is substantially hindered. Next, we vary the persistence time of the self-propelled particles at fixed velocity in the simulations, which cannot be realized experimentally. We then observe a qualitative change in the dynamics of coarsening as a function of the associated persistence length *L*_p_ (Fig. 3). Swimmers with large persistence navigate through the system uniformly and form streaks (Fig. 3b), while self-propelled particles with short persistence co-localize at grain boundaries (Fig. 3a and Supplementary Movie 4), a reminiscence of the effective attraction of hot particles in a cold bath^40^. The melting from the boundaries is analogous to previous numerical results^25,27^ but contrasts with our experimental observations. Further, we study the level of added fluctuations induced by active intruders by measuring the effective diffusivity of the passive spheres *D*_eff_, as a function of *L*_p_ at fixed propulsion. We observe a transition in *D*_eff_ that plateaus for persistence lengths shorter than the passive colloids’ size, $L_{\mathrm{p}}\sim 0.4\sigma$. The qualitative change observed in the dopants’ dynamics (Fig. 3a, b) coincides with a transition in energy transfer between the dopants and the passive particles (Fig. 3c). The co-localization of active particles at the grain boundaries limits the collisions with the passive phase and the level of added noise: the annealing is slowed down. Although the mobility of the grain is conveniently described by an activated process with an effective temperature, the observed dynamical transition highlights the intrinsic non-equilibrium nature of the annealing process.

### Spatial control of the annealing

**Fig. 4:**
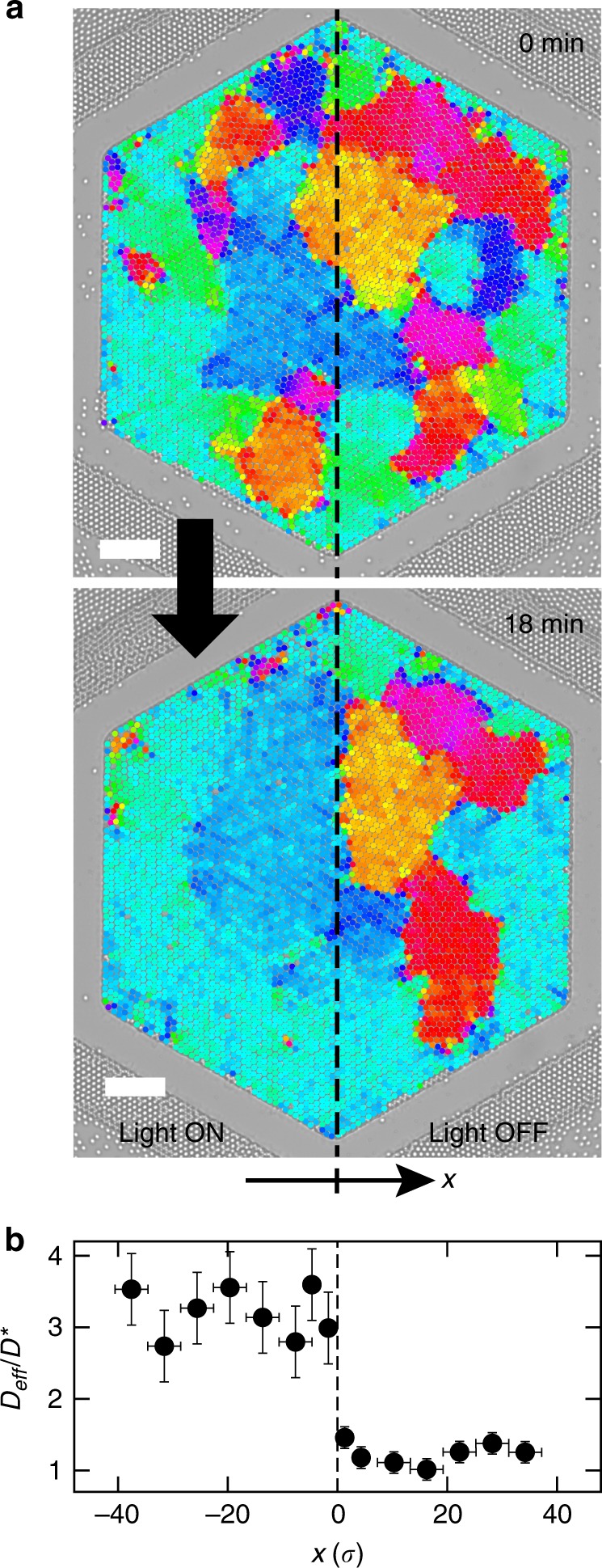
Spatial control of the annealing. **a** Selective reorganization of the polycrystalline layer by activating only the intruders in the left half of the well. Scale bars, 50 μm. **b** Diffusivity of the passive particles *D*_eff_ compared to that in a thermal system D^*^, and plotted as a function of the distance to the illumination interface expressed in units of diameter. It illustrates dynamical control with a spatial resolution of only a few colloids. Vertical errorbars denote the standard deviation over particles located within each spatial window, whose boundaries are shown through horizontal errorbars

We further exploit the potential of activity-driven assembly of colloidal materials by demonstrating spatial control of the annealing in the monolayer. Taking advantage of the flexibility offered by photocatalytic swimmers and light patterns, we selectively activate one half of the colloidal monolayer while keeping the other half in the dark, i.e., in thermal state (Fig. 4a and Supplementary Movie 5). Activity generates fluctuations in the monolayer and passive particles in illuminated regions diffuse many times faster than in dark regions, where *D*_eff_ drops back to its equilibrium value (Fig. 4b). Following, the activated region quickly rearranges, while the thermal region barely evolves, illustrating a dynamical control with a spatial resolution of only a few colloids. It shows the first experimental demonstration of a man-made material controlled by internal activity, regulated in time and space.

## Discussion

Heat treatments, e.g., annealing, quenching, or tempering, were originally developed for metals, to alter macroscopic properties such as strength, ductility, or toughness. Annealing is achieved through cycles of high temperatures and slow cooling: high temperatures steps allow the system to escape kinetic traps, while the slow cooling relaxes the system to its ground state. We showed that active particles embedded in an otherwise passive system offer an untapped approach to annealing. The in situ addition of large fluctuations, mimics macroscopic annealing by heating and locally reorganizing the system. It allows local equilibration and avoids metastable states, a crucial problem in the experimental assembly of complex architectures. We further unveil a transition in the dynamics of annealing, set by the persistence of the active dopants, which has no equilibrium counterpart and had not been predicted in previous numerical works. By using light patterns, we demonstrate a spatial control of the annealing with a spatial resolution of a few microns. The spatiotemporal control of internal fluctuations paves the way to the tuning of material properties, as well as the engineering of bio-inspired sensors harnessing active noise for enhanced accuracy^41,42^. The variety of available synthetic and biological microswimmers, including particles which could remotely be steered by magnetic fields or light patterns^43,44^, offers opportunities for a wide range of control. As grain boundaries are essential to material properties, from yield strength^45,46^ to electrical conductivity^47^, our findings usher materials science to a new age, where the properties of matter are not controlled macroscopically but microscopically and in real-time by active dopants. Further, the use of traveling particles that navigate a matrix as in this work, may suggest innovative numerical protocols of simulated annealing using virtual active intruders rather than global temperature cycling to explore complex energy landscapes.

## Methods

### Experimental set-up

Our colloidal model system consists of silica beads of diameter *σ* = 5 μm (Sigma aldrich, 44054) suspended in a 6% solution of hydrogen peroxide *H*_2_*O*_2_ in deionized water (Millipore, 18.2 MΩ). Within a few minutes, the heavy particles sediment on the bottom wall of the sample cell to form a dense monolayer of area fraction Φ_S_ = *πNσ*^2^/4*A* ≈ 0.68 ± 0.03, where *N* is the number of particle contained in the hexagonal area *A*. The equilibrium gravitational height is much smaller than *σ*, so that out-of-plane thermal fluctuations are negligible in the absence of swimmers and the system is quasi two-dimensional. When active intruders are introduced in the layer, however, passive particle are observed to slightly lift from the bottom surface as smaller swimmers pass by. The numeric fraction of intruders *α* = *N*_s_/*N*, with *N*_s_ the number of swimmers, is varied. They are light-activated 2 μm-diameter particles, consisting of a hematite cube embedded in a polymer bead^20,48^. Under ultraviolet (UV)-light, the photocatalytic hematite triggers the local decomposition of the hydrogen peroxide contained in the solution, creating a gradient that sets the swimmer into motion through phoretic effects. They then exhibit a persistent random walk along the bottom surface.

The sample cell containing the solution is assembled from a glass microscope slide on top and a 0.38 mm-thick polymethylmethacrylate (PMMA) sheet (Goodfellow ME303001) on the bottom, separated by rectangular capillaries (Vitrocom 3524) used as spacers of approximate height 600 μm. Shallow 4 μm-deep hexagonal wells are heat embossed in the PMMA bottom surface using a polydimethylsiloxane (PDMS) mold fabricated via soft lithography. This confinement arena allows to keep the surface fraction Φ_S_ constant for the duration of an experiment. A narrow trench additionally surrounds the hexagon, to trap exterior colloids and prevent them from falling into the well. Quantitative measurements are conducted in 400 μm-wide hexagons, containing about 5600 passive particles. Prior to assembly, all components are washed with Hellmanex III and thoroughly rinsed with deionized water (Milli-Q, resistivity 18.2 M). After injecting the colloidal solution, the cell is sealed with capillary wax (Hampton Research HR4-328).

The system is observed using an inverted optical microscope (Nikon Eclipse-Ti) equipped with a 20× objective. A light source with a wavelength *λ* = 390–480 nm (Lumencor, Spectra X) uniformly illuminates the hexagonal chamber through the bottom wall and activates the swimmers. The intensity of the LED can be adjusted to modify the speed of the intruders. The UV illumination is periodically interrupted for short intervals of 20 s every 80 s to allow for swimmers that are wedged between passive particles and the substrate to reorient through Brownian motion and escape. The evolution of the monolayer is monitored with a camera (Hamamatsu, C11440-22CU) recording images at a frame rate of 2.5 fps for 90 min, and 0.1 fps for 12 h for a thermal system (with no swimmers). The position of the passive particles is extracted in each frame using standard image analysis routines^49^.

### Characterization of the grain structure

The organization of the polycrystalline layer at a time *t* is visualized and quantified through the local orientational bond-order parameter *ψ*_6_. For each passive particle, $\psi _6(\vec r_j,t) = \frac{1}{{N_j}}\mathop {\sum}\nolimits_{j = 1}^{N_j} {e^{6i\theta (\vec r_{jk})}}$ is computed based on the arrangement of its *N*_*j*_ nearest neighbors (defined using Delaunay triangulation), where $\theta (\vec r_{jk})$ is the angle between a particle *j* and its neighbor *k* with respect to a reference axis chosen here as one of the direction of the hexagonal well (see Supplementary Fig. 1b). The amplitude of *ψ*_6_, which takes values within (0.9–1) at the exception of the grain boundaries, reflects local hexagonal order (see Supplementary Fig. 1c). Its phase provides the local crystalline orientation $\theta _6(\vec r_j,t) = \frac{1}{6}{\mathrm{arg}}(\psi _6(\vec r_j,t))$ that varies from 0 to 60° due to rotational symmetry. In Figs. 1, 3, and 4, particles are color-coded with the crystalline orientation to visualize the grain structure. We monitor the evolution of spatial structures using the bond-orientational correlation function *g*_6_(*r*, *t*), calculated from the sixfold bond-order parameter. As previously introduced with thermal systems^32^, the field *ψ*_6_ is first smoothed by averaging local values $\psi _6(\vec r_j,t)$ over the two first shells of neighbors surrounding particle *j*. The resulting field is then normalized, both operations allowing for a more accurate probing of the correlations at short distances^33,50^. The bond-orientational correlation function is then computed as $g_6(r,t) = {\mathrm{Re}}\left( {\left\langle {\hat \psi _6^ \ast (\vec r + \vec r_0,t)\hat \psi _6(\vec r_0,t)} \right\rangle } \right),$ with $\hat \psi _6$ the smoothed and normalized parameter and 〈.〉 referring to the average over all pairs of particles separated by a distance *r*. This definition of *g*_6_(*r*, *t*) evaluates the spatial correlation in the argument of the bond-orientation parameter, singling out the angle component of the long-range order developing in the system over time.

### Numerical simulations

We performed Brownian dynamics simulations using HOOMD-blue^38,39^ to model the experiment. A two-dimensional rectangular box with periodic boundary conditions is filled with 20,000 spheres of diameter *σ* = 1 at an area fraction Φ_S_ = 0.05. A fraction *α* of those particles is randomly selected to form the active particles subset. Their diameter is set to *σ*_s_ = 0.2. All the particles interact through a purely repulsive WCA potential given by:

1 $$U(r) = 4\varepsilon \left[ {\left( {\frac{\xi }{r}} \right)^{2n} - \left( {\frac{\xi }{r}} \right)^n + 1/4} \right]\,{\mathrm{for}}\,r \le 2^{1/n}\xi ,$$

2 $$U(r) = 0\,{\mathrm{for}}\,r > 2^{1/n}\xi ,$$

where *n* = 6, *ε* = 10, and *ξ* = *R*_*i*_ + *R*_*j*_, with *R*_*i*,*j*_ the radius of the interacting particles. *R*_*i*,*j*_ = *σ*/2 for passive particles and *R*_*i*,*j*_ = *σ*_s_/2 for active particles.

The initial configurations are prepared by compressing the diluted box until an area fraction Φ_S_ = 0.67 in 4.10^5^ steps of length Δ*t* = 0.0001 using the Langevin dynamics integrator. At all times, the box dimensions are set to accommodate a hexagonal lattice (i.e., $L_x = L_y \times \sqrt 3$). During this preparation step, the temperature is linearly decreased from *kT* = 2 to 0.0005. Once quenched, the particles form a polycrystalline two-dimensional layer.

Thermal annealing runs are performed by letting the system relax for 500 million steps without adding activity to the small particles. For boosted annealing runs, an active force is applied to the small particles. It is parametrized by its amplitude *F*_a_ and a rotational diffusion constant *D*_R_ that controls the random change of direction of the active force. Typical values for *F*_a_ are between 0 and 4 and shown here in the range (0–1.5), and *D*_R_ is taken between 0.1 and 5. Simulations of activated monolayers are run for 100 million steps. The equations of motion for the simulations are given by:

3 $$m\frac{{\mathrm{d}{\mathbf{v}}}}{{\mathrm{d}t}} = {\mathbf{F}}_{\mathrm{C}} - \zeta \cdot {\mathbf{v}} + {\mathbf{F}}_{\mathrm{r}}$$

4 $$\left\langle {{\mathbf{F}}_{\mathrm{r}}} \right\rangle = {\bf{0}}$$

5 $$\left\langle {\left| {{\mathbf{F}}_{\mathrm{r}}} \right|^2} \right\rangle = 2dkT\zeta /\delta t$$

where **F**_C_ is the force applied on the particles originated from all potentials and constraint forces, *ζ* is the drag coefficient (*ζ* = 1 here), ***v*** is the particle’s velocity, **F**_r_ is a uniform random force and *d* is the dimensionality of the system (*d* = 2 here).

For active particles, an active force is added to the equation of motion such that *δ***r**_*i*_ = *δtF*_a_**p**_i_, where *F*_a_ is the active velocity and **p**_i_ = (cos *θ*_*i*_, sin *θ*_*i*_) is the active force vector for the particle i. The rotational diffusion of this active force vector follows $\delta \theta /\delta t = \sqrt {2D_{\mathrm{R}}/\delta t} {\mathrm{\Gamma }}$, where *D*_R_ is the rotational diffusion constant and the gamma function Γ is a unit-variance random variable that decorrelates space, time, and particles.

The particles positions are recorded every 10,000 steps. Following the same treatment as the experiments, the dynamic of the passive colloids and the swimmers is analyzed. For each set of parameters, six independent runs are performed to improve statistics.

## Supplementary information

Supplementary Information file

Description of Additional Supplementary Files

supplementary movie 1

supplementary movie 2

supplementary movie 3

supplementary movie 4

supplementary movie 5

## Data Availability

The data that support the plots within this paper and other findings of this study are available from the corresponding author upon request.

## Code availability

The code that support the plots within this paper and other findings of this study are available from the corresponding author upon request.
