# Supplementary material for: Activity-controlled annealing of colloidal monolayers
Source: Nat Commun. 2019 Jul 29;10:3380. doi: 10.1038/s41467-019-11362-y (PMC6662715; doi:10.1038/s41467-019-11362-y)
Supplement: Supplementary file 1 — Supplementary Information file [file 41467_2019_11362_MOESM1_ESM.pdf]

Supplementary Information
Activity-controlled Annealing of Colloidal Monolayers

by S. Ramananarivo *et al*

1 Supplementary Note 1: Experimental set-up

Our colloidal model system consists in silica beads of diameter $\sigma = 5\mu m$ (Sigma aldrich, 44054) suspended in a 6% solution of hydrogen peroxide H_2O_2 in deionized water (Millipore, $18.2M\Omega$). Within a few minutes, the heavy particles sediment on the bottom wall of the sample cell to form a dense monolayer of area fraction $\Phi_S = \pi N\sigma^2/4A \approx 0.68 \pm 0.03$, where N is the number of particle contained in the hexagonal area A [Supplementary-Fig.1a]. The equilibrium gravitational height is much smaller than σ, so that out-of-plane thermal fluctuations are negligible in the absence of swimmers and the system is quasi two-dimensional. When active intruders are introduced in the layer, passive particles are however observed to slightly lift from the bottom surface as smaller swimmers pass by. The numeric fraction of intruders $\alpha = N_s/N$, with N_s the number of swimmers, is varied. They are light activated 2μm-diameter particles, consisting in a hematite cube embedded in a polymer bead [1, 2]. Under UV-light, the photo-catalytic hematite triggers the local decomposition of the hydrogen peroxide contained in the solution, creating a gradient that sets the swimmer into motion through phoretic effects. They then exhibit a persistent random walk along the bottom surface.

The sample cell containing the solution is assembled from a glass microscope slide on top and a 0.38mm-thick polymethylmethacrylate PMMA sheet (Goodfellow ME303001) on the bottom, separated by rectangular capillaries (Vitrocom 3524) used as spacers of approximate height 600μm. Shallow 4μm deep hexagonal wells are heat embossed in the PMMA bottom surface using a polydimethylsiloxane PDMS mold fabricated via soft lithography [Supplementary-Fig.1a]. This confinement arena allows to keep the surface fraction Φ_S constant for the duration of an experiment. A narrow trench additionally surrounds the hexagon, to trap exterior colloids and prevent them from falling into the well. Quantitative measurements are conducted in 400μm-wide hexagons, containing about 5600 passive particles. Prior to assembly, all components are washed with Hellmanex III and thoroughly rinsed with deionized water (Milli-Q, resistivity 18.2 M). After injecting the colloidal solution, the cell is sealed with capillary wax (Hampton Research HR4-328).

The system is observed using an inverted optical microscope (Nikon Eclipse-Ti) equipped with a 20× objective. A LED with a wavelength $\lambda = 390 - 480nm$ (Lumencor, Spectra X) uniformly illuminates the hexagonal chamber through the bottom wall and activates the swimmers. The intensity of the LED can be adjusted to modify the speed of the intruders. The UV illumination is periodically interrupted for short intervals of 20s every 80s to allow for swimmers that are wedged between passive particles and the substrate to reorient through Brownian motion and escape. The evolution of the monolayer is monitored with a camera (Hamamatsu, C11440-22CU) recording images at a frame rate of 2.5fps for 90 min, and 0.1fps for 12h for a thermal system (with no swimmers). The position of

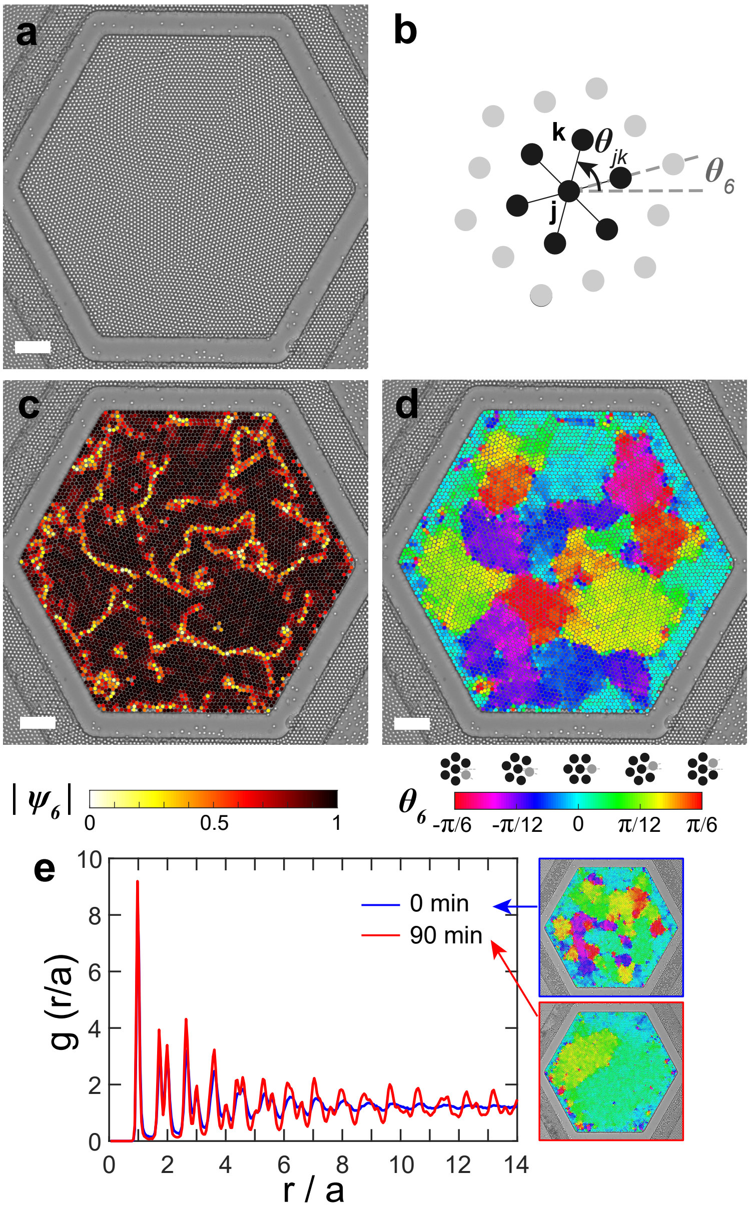

Supplementary Figure 1: **Colloidal system.** **(a)** Microscopy image of a monolayer of $5\mu m$ colloids formed through sedimentation on the bottom surface of the sample cell. The colloidal system is confined within a shallow hexagonal well imprinted in the substrate. **(b)** Arrangement of the nearest neighbors around a particle j characterized by θ_{jk}, the angle of the bond $j-k$ (with k one of its neighbors) with respect to a reference axis. This arrangement is used to compute the six-fold bond order parameter ψ_6 and the local orientation θ_6 (see text). **(c)** Particules are color-coded with the local amplitude $|\psi_6|$, showing hexatic order $|\psi_6| > 0.9$, across the system with the exception of the grain boundaries. **(d)** Particules color-coded with the local orientation θ_6, highlighting the distinct alignements of separate grains. Scale bars, $50\mu m$. **(d)** Radial distribution function $g(r)$ in the initial polycristalline state (blue curve, top image) and in the final ordered one (red curve, bottom image) showing the development of a long-range hexatic order in the system. The relative distance r is normalized by the lattice constant a = 5.4μm, that is determined by the position of the first peak.

the passive particles is extracted in each frame using standard routines [3].

2 Supplementary Note 2: Dynamics of passive colloids

2.1 Characterization of the grain structure

The organization of the polycrystalline layer at a time t is visualized and quantified through the local orientational bond order parameter ψ_6. For each passive particle,

$$\psi_6(\vec{r}_j, t) = \frac{1}{N_j} \sum_{j=1}^{N_j} e^{6i\theta(\vec{r}_{jk})} \tag{1}$$

is computed based on the arrangement of its N_j nearest neighbors (defined using Delaunay triangulation), where $\theta(\vec{r}_{jk})$ is the angle between a particle j and its neighbor k with respect to a reference axis chosen here as one of the direction of the hexagonal well [Supplementary-Fig.1b]. The amplitude of ψ_6, which takes values within [0.9 1] at the exception of the grain boundaries, reflects local hexagonal order [Supplementary-Fig.1c]. This ordered arrangement is also captured by the radial distribution function $g(r)$ that represents the probability to find a colloid a distance r of another colloid, and plotted in [Supplementary-Fig.1e] at the beginning and end of an experiment. The first distinct peaks reflect short-range hexagonal order in both instances, while quasi-long range order develops in time as the polycrystalline layer rearranges.

The phase of ψ_6 further provides the local crystalline orientation

$$\theta_6(\vec{r}_j, t) = \frac{1}{6}\arg(\psi_6(\vec{r}_j, t)) \tag{2}$$

that varies from 0 to 60 due to rotational symmetry. Particles are color-coded with the crystalline orientation to visualize the grain structure in Supplementary-Fig.1d, as well as Fig.1, Fig.3, and Fig.4 of the main text.

We monitor the evolution of spatial structures using the bond-orientational correlation function $g_6(r,t)$, calculated from the six-fold bond-order parameter. As previously introduced with thermal systems [4], the field ψ_6 is first smoothed by averaging local values $\psi_6(\vec{r}_j, t)$ over the two first shells of neighbors surrounding particle j. The resulting field is then normalized, both operations allowing for a more accurate probing of the correlations at short distances [5, 6]. The bond-orientational correlation function is then computed as

$$g_6(r,t) = \mathrm{Re}\left(\left\langle \hat{\psi}_6^{\star}(\vec{r} + \vec{r}_0, t)\hat{\psi}_6(\vec{r}_0, t)\right\rangle\right), \tag{3}$$

with $\hat{\psi}_6$ the smoothed and normalized parameter and $\langle . \rangle$ referring to the average over all pairs of particles separated by a distance r. This definition of $g_6(r,t)$ evaluates the spatial correlation in the argument of the bond-orientation parameter, singling out the angle component of the long-range order developing in the system over time. The inset of Supplementary-Fig.2a shows $g_6(r,t)$ that is ensemble averaged over 5 independent experiments.

Similarly to [6, 7, 4], we then define the characteristic grain size $R_6(t)$ such that $g_6(R_6, t) = 0.5$, as a lengthscale representative of the polycrystalline structure. The collapse of the data that is observed when plotting $g_6(r,t)$ as a function of the dimensionless distance r/R_6 [Supplementary-Fig.2a] corroborates the hypothesis of a dynamical rescaling $g_6(r,t) = f(r/R_6(t))$, with f a time-independent

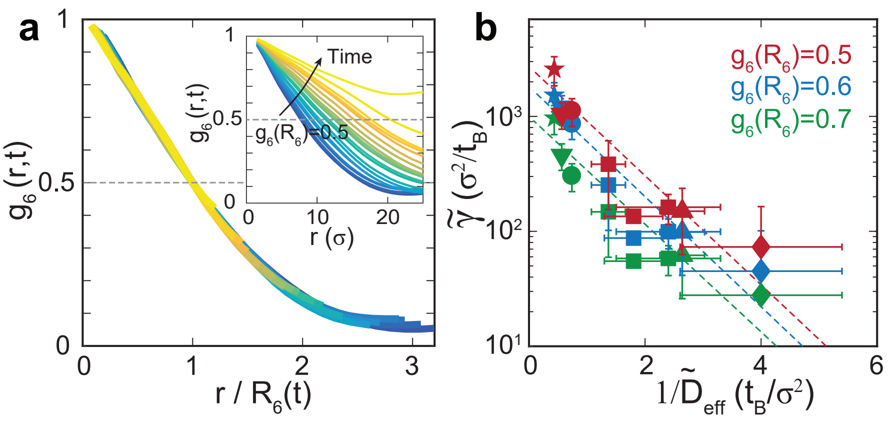

Supplementary Figure 2: **Characteristic grain size.** **(a)** Bond-orientational correlation function $g_6(r,t)$ as a function of the distance r rescaled by the characteristic grain size $R_6(t)$ defined as $g_6(R_6,t) = 0.5$ (see a-inset), for an activated monolayer ($\alpha = 1.3\%$, $V = 10\mu m.s^{-1}$). The collapse of the data reflects the dynamic rescaling of the ordering process, with a self-similar grain structure captured by the lengthscale R_6. **(b)** Grain mobility γ computed for different criteria values for R_6, as a function of the inverse of the diffusion coefficient $1/D_{\text{eff}}$ (see main text). Symbols are introduced in the main text and a single color is used here for all swimming speeds for better visibility. The different definitions only modify γ by a proportionality constant, retaining an Arrhenius-like behavior with a similar activation energy.

function [8, 9]. This emphasizes R_6 as the relevant lengthscale for the system, the crystalline structure then being self-similar once distances are rescaled by this characteristic length. As further discussed below, redefining R_6 by changing the value of the criteria on the right-hand side of $g_6(R_6,t) = 0.5$ does not affect our results and gives a grain time-evolution that is proportional to the former one.

The increase in time of R_6 is reasonably captured by a normal grain growth law $\frac{dR_6}{dt} = \frac{\gamma}{R_6}$, associated to a coarsening mechanism driven by the curvature of the grain boundaries [10, 11, 12]. The parameter γ depends on the mobility and stiffness of the grain boundaries and characterizes their migration rate. It is obtained here from a fit of the data by the power law $R_6^2(t) = R_6^2(0) + \gamma t$ over the first 40min, and we use it to quantify the time-evolution of the system. We however note a departure from a $t^{1/2}$ power law with decreasing intruders activity, that is reminiscent of the variations in growth laws observed for thermal systems due to multiple concurrent effects involved in the coarsening processes [13, 14, 15, 12, 16, 4]. Note that reasonable modifications of the threshold for the determination of R_6 do not affect our results of an activated annealing process. As mentioned above, this only multiply the growth law parameter γ by a proportionality constant, but data retain an Arrhenius-like behavior $\gamma \propto \exp - A/D_{\text{eff}}$ (see Main Text), with a similar energy barrier derived from the slope A [Supplementary-Fig.2b].

2.2 Diffusivity of colloids within the monolayer

We monitor the diffusion of the passive colloids within the crystalline layer. The mean square displacement of all particles is computed over the first 20 min of an experiment when most of the rearrangement occurs. Data are further ensemble averaged over independent experiments; $\langle . \rangle$ will further denotes the ensemble average over all particles and experimental runs. Supplementary-Fig.3a shows the resulting $\langle \Delta r^2 \rangle$ in time for varying speeds and fractions of swimmers. Particles exhibit a long-time diffusive dynamic with the mean square displacement increasing linearly in time. We characterize this behavior with an effective diffusion coefficient D_{eff} measured from a linear fit $\langle \Delta r^2 \rangle = 4D_{\text{eff}}\Delta t$ over 4min. Activated layers exhibit a distinct diffusional enhancement with respect to thermal ones (black

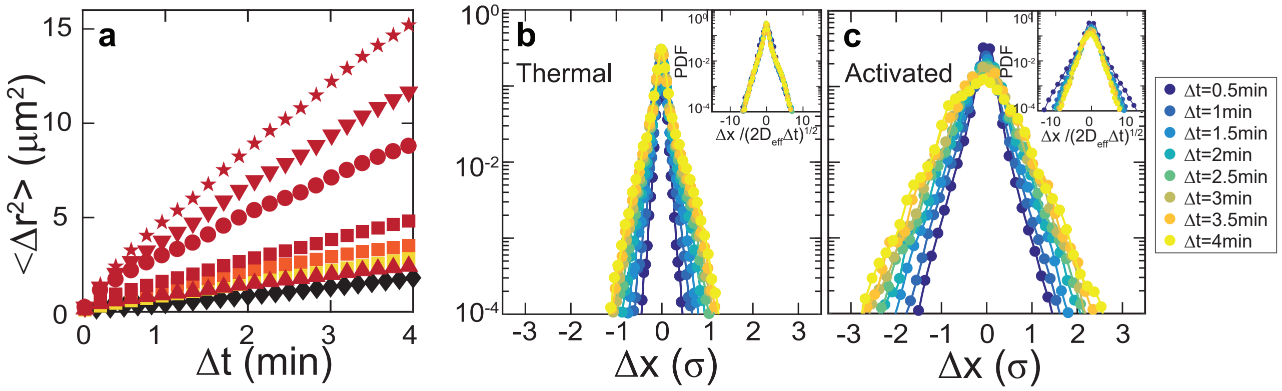

Supplementary Figure 3: **Diffusion of the passive beads of the monolayer (experiment) (a)** Mean square displacement of the passive colloids within the monolayer for different speeds and fractions of active particles, showing a long-time diffusive behavior. Symbols and colors are introduced in the main text: colors distinguish speeds V = 3 (yellow), 5 (orange), $10\mu m.s^{-1}$ (dark red); and symbols refer to different fraction of swimmers α = 0.4 (upward triangle), 1.3 (square), 3.1 (circle), 4.1 (downward triangle), and 5.0% (star). Black is the thermal system, in the absence of activity. **(b)** Probability distribution function of the colloids displacement Δx along an arbitrary axis at various time intervals, in the absence of swimmers, and **(c)** with activity (α = 1.3%, V = $10\mu m.s^{-1}$). The collapse of the data in the insets shows diffusive rescaling of the PDF.

diamond).

Supplementary-Fig.3 further show the probability distribution function of colloids displacements Δx along an arbitrary axis and at different time intervals within a thermal (b) and an activated (c) monolayer. The broader PDFs of Supplementary-Fig.3c reflect the large steps made by the passive beads colliding with the microswimmers. The PDFs further exhibit self-similarity at longer times under a diffusive rescaling when expressing the displacement as $\Delta x/\sqrt{2D_{\text{eff}}\Delta t}$ [insets]. This self-similarity does not hold for activated monolayers at small Δt [Supplementary-Fig.3c, inset], which reflects the additional time-scale introduced by the presence of moving intruders at short times.

3 Supplementary Note 3: Dynamics of swimmers

Due to their smaller size compared to the surrounding colloidal background, the swimmers are difficult to detect robustly through standard particle detection procedures. They can however be located through the distinct empty wake they create as they navigate through the colloidal layer [Supplementary-Fig.4a]. A custom image analysis program thus identify the opening created by a moving swimmers between two successive frames to detect its position. A median filter is first applied to the images to smooth out the outlines of the passive colloids. The substraction of two successive frames then yields bright spots in the places where moving swimmers have displaced colloids, that are smoothed with a Gaussian filter to remove noise. Those spots are then detected through standard routines [3], and provide the position of the swimmers with an accuracy of $\pm 2\mu$m.

Individual trajectories are then reconstructed. Swimmers exhibit a persistent random walk: they travel in straightline along the directions of the crystal with a speed V and their direction is randomized over a persistence time τ_{R}. Their dynamics is captured by the Langevin equation that expresses their mean square displacement in time [17, 18]:

$$\langle \Delta r^2 \rangle = 4D_{\text{s}}\Delta t + \frac{V^2\tau_{\text{R}}^2}{2}\left[\frac{2\Delta t}{\tau_{\text{R}}} + e^{-2\Delta t/\tau_{\text{R}}} - 1\right], \qquad (4)$$

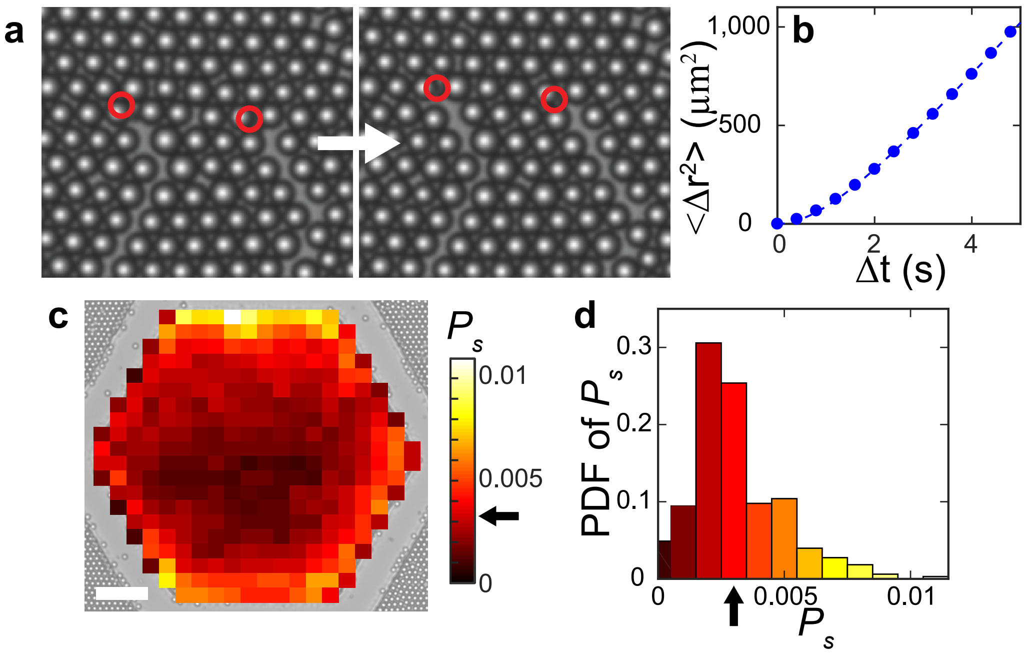

Supplementary Figure 4: **Dynamics of swimmers.** **(a)** Motion of active particles in between two successives frames, whose locations (red circles) are determined through the empty wake created as they navigate through the colloidal monolayer. **(b)** Mean square displacement of swimmers under maximal UV illumination, fitted by a Langevin equation (dotted line, see text), yielding a swimming speed $V = 10\mu m.s^{-1}$ and persistence time $\tau_R = 2s$. **(c)** Probability distribution P_s of the presence of swimmers in the colloidal layer over the first 20min of an experiment. Scale bar, $50\mu m$. **(d)** PDF of P_s showing that most spatial windows have values of P_s corresponding to that of a uniform distribution of swimmers (indicated by an arrow).

with D_s the diffusion coefficient of the swimmers at equilibrium (no UV light). A value of $D_s = 0.3 \pm 0.1\mu m^2.s^{-1}$ is extracted from independent experiments. Fitting the mean square displacement curve averaged over about 50 individual swimmers and over an interval of 60s [Supplementary-Fig.4b], we obtain the speed and the persistence time that reaches $V = 10\mu m.s^{-1}$ and $\tau_R = 2s$ for maximal UV illumination. Speeds of $3\mu m.s^{-1}$ and $5\mu m.s^{-1}$ are additionally achieved by respectively reducing the light to 5% and 20% of its maximum intensity. Swimmers maintain a constant speed throughout an experiment. They explore all regions of the colloidal well with fairly equal probability, resulting in a uniform spatial distribution [Supplementary-Fig.4c and d]. We record a slow decrease in the number of swimmers due to a higher proportion of swimmer leaving the illuminating the hexagonal arena than re-entering it, and therefore limit our observations to the first 60 mins of the experiment where the number of swimmers is reasonably constant.

4 Supplementary Note 4: Numerical simulations

We performed Brownian dynamics simulations using HOOMD-blue [19, 20] to model the experiment. A two-dimensional rectangular box with periodic boundary conditions is filled with 20 000 spheres of diameter $\sigma = 1$ at an area fraction $\Phi_S = 0.05$. A fraction α of those particles is randomly selected to form the active particles subset. Their diameter is set to $\sigma_s = 0.2$. All the particles interact through

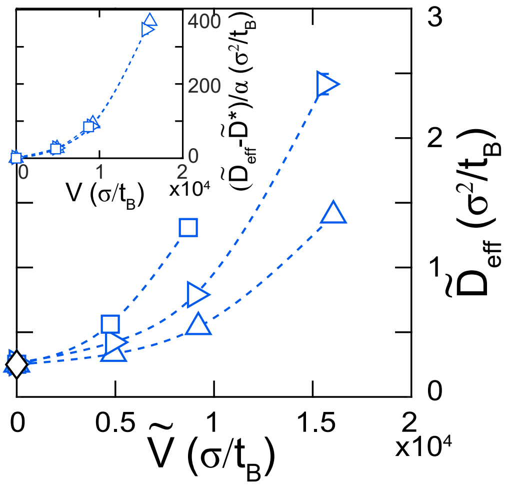

Supplementary Figure 5: **Diffusion of passive particles in doped monolayers (simulation).** Diffusivity of passive particles $\tilde{D}_{\text{eff}}$ in simulations as a function of the speed of the active intruders $\tilde{V}$, with data nondimensionalized using σ and the Brownian time t_B as a characteristic length and time (see Main text). Symbols differentiate between fractions of swimmers α = 0.3 (△), 0.6 (▷), and 1.2% (□); with the black diamond referring to thermal systems. **(inset)** Difference between $\tilde{D}_{\text{eff}}$ and the diffusivity of thermal systems $\tilde{D}^*$, showing a collapse of data when rescaled by α. Errorbars indicate the standard deviation of the different runs (non visible when smaller than the symbol size).

a purely repulsive WCA potential given by :

$$U(r) = 4\epsilon\left[\left(\frac{\xi}{r}\right)^{2n} - \left(\frac{\xi}{r}\right)^{n} + 1/4\right] \text{ for } r \leq 2^{1/n}\xi, \quad (5)$$

$$U(r) = 0 \text{ for } r > 2^{1/n}\xi, \quad (6)$$

where $n = 6$, $\epsilon = 10$ and $\xi = R_i + R_j$, with $R_{i,j}$ the radius of the interacting particles. $R_{i,j} = \sigma/2$ for passive particles and $R_{i,j} = \sigma_s/2$ for active particles.

The initial configurations are prepared by compressing the diluted box until an area fraction $\Phi_S = 0.67$ in 4.10^5 steps of length $\Delta t = 0.0001$ using the Langevin dynamics integrator. At all times, the box dimensions are set to accommodate a hexagonal lattice (i.e. $L_x = L_y \times \sqrt{3}$). During this preparation step, the temperature is linearly decreased from $kT = 2$ to 0.0005. Once quenched, the particles form a polycrystalline 2D layer.

Thermal annealing runs are performed by letting the system relax for 500 million steps without adding activity to the small particles. For boosted annealing runs, an active force is applied to the small particles. It is parametrized by its amplitude F_a and a rotational diffusion constant D_R that controls the random change of direction of the active force. Typical values for F_a are between 0 and 4 and shown here in the range [0-1.5], and D_R is taken between 0.1 and 5. Simulations of activated monolayers are run for 100 million steps.

The equations of motion for the simulations are given by :

$$m\frac{d\mathbf{v}}{dt} = \mathbf{F}_C - \zeta \cdot \mathbf{v} + \mathbf{F}_r \quad (7)$$

$$\langle \mathbf{F}_r \rangle = \mathbf{0} \quad (8)$$

$$\langle |\mathbf{F}_r|^2 \rangle = 2dkT\zeta/\delta t \quad (9)$$

where $\mathbf{F}_C$ is the force applied on the particles originated from all potentials and constraint forces, ζ is the drag coefficient ($\zeta = 1$ here), $\mathbf{v}$ is the particle's velocity, $\mathbf{F}_r$ is a uniform random force and d is the dimensionality of the system ($d = 2$ here).

For active particles, an active force is added to the equation of motion such that $\delta \mathbf{r}_i = \delta t F_a \mathbf{p}_i$, where F_a is the active velocity and $\mathbf{p}_i = (\cos\theta_i, \sin\theta_i)$ is the active force vector for the particle i. The rotational diffusion of this active force vector follows $\delta\theta/\delta t = \sqrt{2D_R/\delta t}\Gamma$, where D_R is the rotational

diffusion constant and the gamma function Γ is a unit-variance random variable that decorrelates space, time and particles.

The particles positions are recorded every 10000 steps. For each set of parameters, six independent runs are performed to improve statistics. Following the same treatment as the experiments, the dynamic of the passive colloids is analyzed as presented in Supplementary Note 2 in both presence or absence of active particles. The swimmers' dynamics is also characterized following Supplementary Note 3. Supplementary-Fig.5 shows the diffusivity of passive particles $\tilde{D}_{\text{eff}}$ as a function of the measured velocity of active particles $\tilde{V}$. As for experiment, the diffusion within the crystalline layer is enhanced by the activity of its intruders, and the collapse in the inset confirms that this enhancement scales with the fraction of swimmers in the considered regime of very few intruders. The increase of $\tilde{D}_{\text{eff}}$ with $\tilde{V}$ is however faster than the linear dependency observed in experiments. This deviation owes to differences in transmission of momentum from active to passive particles, in between simulations and experiments. Simulations do not account for hydrodynamics or the dissipation associated to the presence of a substrate. Additionally, the two-dimensional nature of simulations confines momentum transfers within a plane, whether passive colloids are observed to slightly lift from the bottom surface as swimmers pass by in experiments. However, the dynamics of annealing agree in simulations and in experiment when considering $\tilde{\gamma}(\tilde{D}_{eff})$ the effect of the enhancement of effective diffusion on the annealing of the monolayer [see Main Text].

Supplementary References

[1] J. Palacci, S. Sacanna, A. P. Steinberg, D. J. Pine, and P. M. Chaikin. Living crystals of light-activated colloidal surfers. *Science*, page 1230020, 2013.

[2] J. Palacci, S. Sacanna, S-H. Kim, G-R. Yi, D. J. Pine, and P. M. Chaikin. Light-activated self-propelled colloids. *Phil. Trans. R. Soc. A*, 372(2029):20130372, 2014.

[3] D. Blair and E. Dufresne. *Matlab particle tracking code retrieved from http://physics.georgetown.edu/matlab/.*

[4] F. A. Lavergne, D. G. A. L. Aarts, and R. P. A. Dullens. Anomalous grain growth in a polycrystalline monolayer of colloidal hard spheres. *Phys. Rev. X*, 7(4):041064, 2017.

[5] R. E. Blundell and A. J. Bray. Phase-ordering dynamics of the o (n) model: Exact predictions and numerical results. *Phys. Rev. E*, 49(6):4925, 1994.

[6] A. Sicilia, J. J. Arenzon, I. Dierking, A. J. Bray, L. F. Cugliandolo, J. Martínez-Perdiguero, I. Alonso, and I. C. Pintre. Experimental test of curvature-driven dynamics in the phase ordering of a two dimensional liquid crystal. *Phys. Rev. Lett.*, 101(19):197801, 2008.

[7] M. P. O. Loureiro, J. J. Arenzon, L. F. Cugliandolo, and A. Sicilia. Curvature-driven coarsening in the two-dimensional potts model. *Phys. Rev. E*, 81(2):021129, 2010.

[8] A. J. Bray. Theory of phase-ordering kinetics. *Adv. Phys.*, 51(2):481–587, 2002.

[9] L. F. Cugliandolo. Topics in coarsening phenomena. *Physica A Stat. Mech. Appl.*, 389(20):4360–4373, 2010.

[10] S. M. Allen and J. W. Cahn. A microscopic theory for antiphase boundary motion and its application to antiphase domain coarsening. *Acta Metall.*, 27(6):1085–1095, 1979.

[11] G. Gottstein and L. S. Shvindlerman. *Grain boundary migration in metals: thermodynamics, kinetics, applications*. CRC press, 2009.

[12] A. Rollett, F. J. Humphreys, G. S. Rohrer, and M. Hatherly. *Recrystallization and related annealing phenomena*. Elsevier, 2004.

[13] G. T. Higgins. Grain-boundary migration and grain growth. *Met. Sci.*, 8(1):143–150, 1974.

[14] P. A. Deymier, J. O. Vasseur, and L. Dobrzynski. Anomalous exponent in the kinetics of grain growth with anisotropic interfacial energy. *Phys. Rev. B*, 55(1):205, 1997.

[15] C. Harrison, D. E. Angelescu, M. Trawick, Z. Cheng, D. A. Huse, P. M. Chaikin, D. A. Vega, J. M. Sebastian, R. A. Register, and D. H. Adamson. Pattern coarsening in a 2d hexagonal system. *Europhys. Lett.)*, 67(5):800, 2004.

[16] D. A. Vega, C. K. Harrison, D. E. Angelescu, M. L. Trawick, D. A. Huse, P. M. Chaikin, and R. A. Register. Ordering mechanisms in two-dimensional sphere-forming block copolymers. *Phys. Rev. E*, 71(6):061803, 2005.

[17] J. R. Howse, R. A. L. Jones, A. J. Ryan, T. Gough, R. Vafabakhsh, and R. Golestanian. Self-motile colloidal particles: from directed propulsion to random walk. *Phys. Rev. Lett.*, 99(4):048102, 2007.

[18] J. Palacci, C. Cottin-Bizonne, C. Ybert, and L. Bocquet. Sedimentation and effective temperature of active colloidal suspensions. *Phys. Rev. Lett.*, 105(8):088304, 2010.

[19] J. A. Anderson, C. D. Lorenz, and A. Travesset. General purpose molecular dynamics simulations fully implemented on graphics processing units. *J. Comput. Phys.*, 227(10):5342–5359, 2008.

[20] J. Glaser, T. D. Nguyen, J. A. Anderson, P. Lui, F. Spiga, J. A. Millan, D. C. Morse, and S. C. Glotzer. Strong scaling of general-purpose molecular dynamics simulations on gpus. *Comput. Phys. Commun.*, 192:97–107, 2015.
