# Supplementary material for: Activity-controlled annealing of colloidal monolayers
Source: Nat Commun. 2019 Jul 29;10:3380. doi: 10.1038/s41467-019-11362-y (PMC6662715; doi:10.1038/s41467-019-11362-y)
Supplement: Supplementary file 2 — Description of Additional Supplementary Files [file 41467_2019_11362_MOESM2_ESM.pdf]

Description of supplementary Files

Supplementary movie 1: Colloidal monolayer with active dopants. Video microscopy of colloidal monolayers of passive beads in a hexagonal chamber of width 260 µm. The left one contains a small fraction of self-propelled colloids (α=1.2%, V=10 µm/s), while the right one is purely passive. Although the swimmers are difficult to distinguish due to their smaller size, they can be localized by their wake as they navigate the colloidal monolayer. The addition of active particles massively accelerates the annealing of the polycrystalline monolayer that rapidly converts into an ordered crystal, while its thermal counterpart evolves much slower. The movie shows the first 55min of the experiments. It is initially sped up 10 times then accelerated 300 times as indicated.

Supplementary movie 2: Accelerated annealing. Video microscopy of a colloidal monolayer of passive beads in a hexagonal chamber of width 400 µm, and containing a small fraction of active intruders (α=5%, V=10 µm/s) and overlaid with the local crystalline order (colored beads). It highlights the presence of distinct grains. The system coarsens as a large ordered domain develops at the expense of smaller grains. The movie shows the first 35min of the experiment and is sped up 50 times.

Supplementary movie 3: Simulated colloidal layer with active dopants. Two-dimensional Brownian dynamics simulation of a polycrystalline monolayer of 2.10^4 particles with periodic boundary conditions, and containing a fraction α =0.6% of active intruders with persistence length L_p=7σ comparable with the experiment. A zoomed view is shown on the right. Similarly to experiments, the intruders propel through the entire layer following the local directions of the crystal and speed up the annealing of the colloidal monolayer to a long range hexatic order. In the zoomed-out video (left), active particles are shown as black points bigger than their actual size to improve visibility.

Supplementary movie 4: Simulated regimes with various persistence active particles. Two-dimensional Brownian dynamics simulations of polycrystalline monolayers containing a fraction α =0.6% of active intruders with the same speed but different persistence. On the left, the corresponding persistence length L_p~7σ is similar to that of experiments, which produces the regime observed experimentally. On the right, L_p ~ 0.3 σ is shorter and intruders exhibit a significantly different dynamic: they tend to accumulate at grain boundaries where reduced particle density provides lower stiffness or resistance to motion. The latter activated system takes

longer to anneal. The middle part of the movie is sped up 20 times to highlight the different evolution of the crystalline structure. The active particles are shown as black points bigger than their actual size to improve visibility. Particles are color-coded with the local crystalline orientation to better visualize the evolution of the polycrystalline structure.

Supplementary movie 5: Spatial control of the reorganization. Video microscopy of a colloidal monolayer of passive beads in a hexagonal chamber of width 400 µm, and containing a small fraction of active intruders (α=3%, V=10 µm/s). Using a light pattern, we selectively activate the left half of the confinement arena. The video show spatial control of the annealing rate of the monolayer: the activated region reorganizes into one crystal, while the other region evolves on much longer time scales. Particles are color-coded with the local crystalline orientation to better visualize the evolution of the polycrystalline structure. The movie is sped up 50 times and its real time duration is 18min.
